# Ectopic Expression of a Pak-choi YABBY Gene, *BcYAB3*, Causes Leaf Curvature and Flowering Stage Delay in *Arabidopsis thaliana*

**DOI:** 10.3390/genes11040370

**Published:** 2020-03-29

**Authors:** Hualan Hou, Ye Lin, Xilin Hou

**Affiliations:** State Key Laboratory of Crop Genetics & Germplasm Enhancement, Key Laboratory of Biology and Genetic Improvement of Horticultural Crops in East China, Ministry of Agriculture and Rural Affairs of the P. R. China, Engineering Research Center of Germplasm Enhancement and Utilization of Horticultural Crops, Ministry of Education of the P. R. China, Nanjing 210095, China; 2017204021@njau.edu.cn (H.H.); 2018104050@njau.edu.cn (Y.L.)

**Keywords:** *BcYAB3*, leaf development, adaxial–abaxial polarity, Pak-choi, *Arabidopsis*

## Abstract

The YABBY family are a group of seed plant-specific transcription factors, which are involved in the specification of abaxial polarity in lateral organs. In *Arabidopsis thaliana*, *YABBY3* (*YAB3*) plays a critical role in regulating abaxial patterning, growth of lateral organs, and inflorescence phyllotaxy. In this study, the *BcYAB3* gene was isolated from Pak-choi (*Brassica rapa* subsp. *chinensis*). The tissue-specific expression analysis indicated that the *BcYAB3* gene has significantly high transcript levels in stem, leaf, and flower. We investigated the subcellular localization of BcYAB3 and found the protein to be expressed in the nucleus. In the transgenic *Arabidopsis* *thaliana* plants expressing the *BcYAB3* gene, the leaves were curling downward with the plant growth, and the bolting and flowering stages were delayed. These results not only validate the function of *BcYAB3* in the leaf and flower development in *Arabidopsis*, but also contribute to unravel the molecular regulatory mechanism of *YAB3* gene in the establishment of adaxial–abaxial polarity of the lateral organs in Pak-choi.

## 1. Introduction

The leaf is a major organ of plants, responsible for photosynthesis, respiration, and transpiration. Therefore, the flattening of the leaf is essential for maximizing photosynthesis. Leaf shape generally depends on the development of three axes of polarity, namely the proximal–distal axis, adaxial–abaxial axis, and medial–lateral axis. Among them, the adaxial–abaxial axis serve as the basis for establishment of the medial–lateral axis and proximal–distal axis [1]. Moreover, the juxtaposition of adaxial and abaxial cells is essential for normal lamina outgrowth [2].

The adaxial–abaxial polarity is established in the development of lateral organs and controlled by several transcriptional factors and small non-coding RNAs, such as the HD-Zip III family, *AS1*/*AS2* (*ASYMMETRIC LEAVES1/2*), KANADI family, *ARF3*/*4* (*AUXIN RESPONSE FACTOR3/4*), YABBY family, and *miR165*/*166* (*microRNA165*/*166*) [3,4,5,6,7,8]. YABBY genes belong exclusively to the seed plants [9], have a conserved C2C2 zinc finger like domain at the N-terminus, and helix–loop–helix motif analogous to a high mobility group (HMG) box (termed as YABBY domain) at the C-terminus [10,11,12]. In angiosperms, the YABBY family can be divided into five subgroups according to evolutionary analysis and expression profiles of YABBY genes [12,13], namely *FILAMENTOUS FLOWER* (*FIL*)/*YABBY3* (*YAB3*), *YABBY2* (*YAB2*), *INNER NO OUTER* (*INO*), *CRABS CLAW* (*CRC*), and *YABBY5* (*YAB5*). In the previous works, a large number of YABBY genes were identified from different species; they participate in SAM development, establishment of leaf adaxial–abaxial polarity, and lamina and floral organ growth [14,15,16,17,18]. 

In *Arabidopsis*, *FIL*/*YAB3*/*YAB5* are expressed in the abaxial regions of leaves and floral tissues [14,19]. The *fil yab3* double mutant results in ectopic SAM structures, partial loss of abaxial cell identity, and abnormal vasculature, while the ectopic expression of *FIL* and *YAB3* causes partial abaxialization of leaves [14]. In addition, *fil yab3 yab5* triple and *fil yab2 yab3 yab5* quadruple mutants exhibit dramatic polarity defects of lateral organs and loss of lamina expansion [11]. In *Antirrhinum majus*, the mutation of *GRAMINIFOLIA* (*GRAN*) gene, ortholog of *FIL* in *Arabidopsis*, leads to significant adaxialization along the abaxial margin of leaves [15]. In *Solanum lycopersicum*, *LeYAB2* (a *YABBY2*-like gene) is involved in the development of pericarp cells in the abaxial region [20,21,22]. These results illustrate that YABBY genes play a pivotal role in defining the abaxial identity of lateral organs in eudicots. However, YABBY genes have different expression patterns in monocots. For example, the *OsYAB1* gene, ortholog of *YAB2* in *Arabidopsis*, has no polar characteristics when expressed in lateral organs [23]. In maize, YABBY genes are expressed in the adaxial sides of the leaves [24]. *TaYAB1*, a wheat YABBY gene, was overexpressed in *Arabidopsis*, the adaxial surface characteristic trend, and the formation of leaf adaxial polarity were affected [25]. These results demonstrated that functional divergence of YABBY genes occurred in monocots and dicots [26].

Pak-choi (*Brassica rapa* subsp. *chinensis*), which belongs to the *Brassica* species of the *Cruciferae* family, is an economically important vegetable and widely cultivated in Asia. Leaves are the main edible organs of Pak-choi. Therefore, it is significant to understand the regulatory mechanism of leaf polarity in Pak-choi. In *Arabidopsis*, *FIL* and *YAB3* proteins are involved in the regulation of inflorescence phyllotaxy, abaxial patterning, and development of lateral organs. The function of the *FIL* homologous gene has been extensively studied in many species [19,27,28,29,30], while a functional role of the *YAB3* gene has not been fully documented. In this study, the *BcYAB3* gene was isolated from Pak-choi, and the transgenic *Arabidopsis* overexpressed *BcYAB3* was acquired using the floral dip method. The ectopic expression of *BcYAB3* induced strong leaf curling and flowering stage delay in *Arabidopsis*. 

## 2. Materials and Methods

### 2.1. Plant Materials and Cultivation Management

In this study, the Pak-choi cultivar “*suzhouqing*” was used. The seeds were scattered in a plastic petri dish with wet filter paper and grown in pots containing an organic matrix, namely a vermiculite (2:1) mixture in phytotron under controlled environmental conditions (16 h light/8 h dark photoperiod at 23 °C/17 °C). The different tissue (leaf, petiole, stem, root, hypocotyl, flower bud, flower bud, pod) samples were collected at seedling, rosette, flowering, and podding stages. *Nicotiana benthamiana* and *Arabidopsis thaliana* wild type (ecotype Col-0) were used in this study and grown in illumination incubators under the same conditions. 

### 2.2. Isolation and Characterization Analysis

Total RNAs were isolated from different tissues using an RNAeasy Mini Kit (Tiangen, Beijing, China), and the first strands of cDNA were synthesized via reverse transcription using a PrimeScript™ II 1st Strand cDNA Synthesis Kit (Takara, Dalian, China). The CDS of *BcYAB3* was amplified using gene-specific primers based on the sequence of Chinese cabbage *BrYAB3* (Bra037320, http://brassicadb.org/brad/index.php) via homology cloning according to a previous report [31]. The PCR product was cloned into the pMD18-T vector and sequenced by GenScript Company (Nanjing, China). The physicochemical characteristics of *BcYAB3* were predicted using the Expasy website (https://web.expasy.org/protparam/). The secondary structure of BcYAB3 was predicted by PSIPRED 4.0 (http://bioinf.cs.ucl.ac.uk/psipred/). 

### 2.3. Sequence Alignment and Phylogenetic Analysis 

The sequences of *Arabidopsis* YABBY proteins were retrieved from the TAIR database (https://www.arabidopsis.org/). The protein sequence data of *Solanum lycopersicum*, *Vitis vinifera*, *Zea mays*, and *Brassica oleracea* were downloaded from Plant-TFDB (http://planttfdb.cbi.pku.edu.cn/index.php). The protein sequences for *Oryza sativa*, *Antirrhinum majus*, *Triticum aestivum*, *Vitis pseudoreticulata*, and Pak-choi were obtained from previous reports [29,32]. Sequencing alignment was obtained using ClustalW software. The phylogenetic analysis was performed using MEGA 7.0 software using the neighbor-joining (NJ) method, and the bootstrap value was set at 1000 replications. All protein sequences used in this study are listed in Table S1. 

### 2.4. Subcellular Localization Analysis

The protein-coding region of *BcYAB3* was introduced into the pRI101-GFP vector digested with BamHI and NdeI restriction enzymes to generate the construct 35S:*BcYAB3*-GFP. Then, the plasmid of fusion construct and empty vector were transformed into *Agrobacterium* GV3101. The epidermal cell transformation of tobacco leaves was carried out with injection of *Agrobacterium*. After dark culture for 24 h, the seedlings were transferred to normal growth for 24–36 h, and then fluorescent pictures were taken using a confocal laser scanning microscope (Zeiss, LSM780, Jena, Germany).

### 2.5. Transformation and Screening of BcYAB3 Transgenic Plants

The *BcYAB3* CDS was subcloned into the binary vector pCAMBIA1301 to generate the 35S:*BcYAB3*–GUS plant expression vector. The recombinant construct was introduced into *Agrobacterium* GV3101 and then transformed into *Arabidopsis thaliana* (Col) using the floral dip method [33]. The seeds of the T_0_ generation were harvested and screened on solid Murashige and Skoog (MS) medium containing 30 mg/L hygromycin. Resistant plants were subjected to further verification using PCR amplification and qRT-PCR experiments. The T_3_ transgenic lines were used for subsequent phenotype observation and functional analysis. The leaf length and leaf width of transgenic and wild type *Arabidopsis thaliana* were measured using a Vernier caliper for 25-day-old seedlings, and 25 plants of each line were analyzed. ANOVA (analysis of variance) was used for statistical analysis. 

### 2.6. Real-Time PCR

Total RNA of Pak-choi and *Arabidopsis* plants was extracted using an RNAeasy Mini Kit (Tiangen, Beijing, China), and the cDNA for real time PCR was synthesized using a PrimeScript™RT reagent Kit with gDNA Eraser (Takara, Dalian, China). qRT-PCR was carried out with SYBR® Premix Ex Taq^TM^ II (Tli RNaseH Plus) (Takara, Dalian, China) using the ABI StepOnePlus™ Real-Time PCR System (Applied Biosystems, Foster City, CA, USA). The PCR procedure was carried out with the following parameters: 95 °C for 30 s, 40 cycles of 95 °C for 5 s, and 60 °C for 30 s. Furthermore, a melting curve was employed to verify the specificity of all reactions. The *Actin* gene (referred to as *Bra028615*) was used as a housekeeping gene to normalize the transcript levels of *BcYAB3* genes among different tissues [34]. *Actin* of *Arabidopsis* was used as an internal control gene, and the transcript levels were calculated using the 2^−ΔΔCt^ method [35]. Three biological and technical replicates were implemented for each reaction. All primers used in this study are listed in Table S2.

## 3. Results 

### 3.1. Phylogenetic Analysis of BcYAB3 

**Figure 1:**
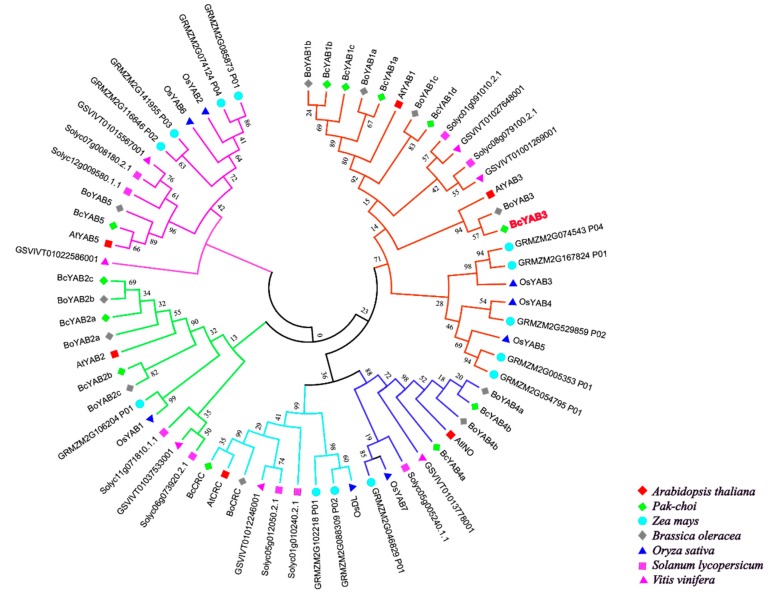
Phylogenetic tree analysis of *BcYAB3*. Phylogenetic relationships of YABBY genes involving with *Arabidopsis thaliana*, *Oryza sativa*, *Zea mays*, *Solanum lycopersicum*, *Vitis vinifera*, *Brassica oleracea*, and Pak-choi. The phylogenetic tree was constructed using the neighbor-joining method, and the bootstrap value was set at 1000.

Until now, hundreds of YABBY genes have been identified in plants. For example, a total of 6 YABBY genes were isolated in *Arabidopsis*, 8 members in rice [36], 9 members in tomato [37], 12 members in Pak-choi [32], 13 members in maize [38], 17 members in soybean [39], et al. In this study, an unrooted phylogenetic tree was constructed based on protein sequences from different species, including two monocots (*Oryza sativa*, *Zea mays*) and five dicots (*Arabidopsis*, *Solanum lycopersicum*, *Vitis vinifera*, *Brassica oleracea*, Pak-choi) (Figure 1, Table S1). Several lines of evidence have shown that YABBY family members can be divided into five groups [9,13,36,40]. As shown in the tree, Pak-choi YABBY genes are distributed in five subclasses, and most YABBY genes from dicots are clustered together and separated from monocots, suggesting that they have functional differentiation. Therefore, the functions of Pak-choi YABBY genes could be deduced from previous studied YABBYs via phylogenetic relationships.

### 3.2. Characterization and Expression Profile of BcYAB3 Gene in Pak-choi

**Figure 2:**
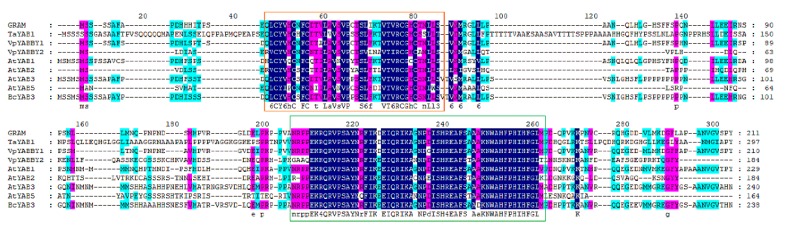
Multiple sequence alignment of *BcYAB3*. The *BcYAB3* gene was aligned with YABBY genes and have been reported to be in *Antirrhinum majus*, *Triticum aestivum*, *Vitis pseudoreticulata*, and *Arabidopsis thaliana*. The conserved C2C2 zinc finger domain and YABBY domain are presented using an orange box and green box, respectively.

The *BcYAB3* gene was isolated from Pak-choi using the homology cloning method. The full-length fragment of *BcYAB3* contained a 717 bp coding sequence encoding 238 amino acid residues. Expasy online prediction indicated that the BcYAB3 protein is an unstable hydrophilic protein (GRAVY = −0.432); the molecular weight is 26.21 KDa, and theoretical isoelectric point (pI) is 8.66. The BcYAB3 protein has a highly conserved domain of the YABBY family at the N-terminal (20–198 amino acid sites), containing C2C2 zinc finger and HMG domains. In addition, secondary structure analysis demonstrated that the BcYAB3 protein is composed of a strand, helix, and coil (Figure 2, Figure S1). 

**Figure 3:**
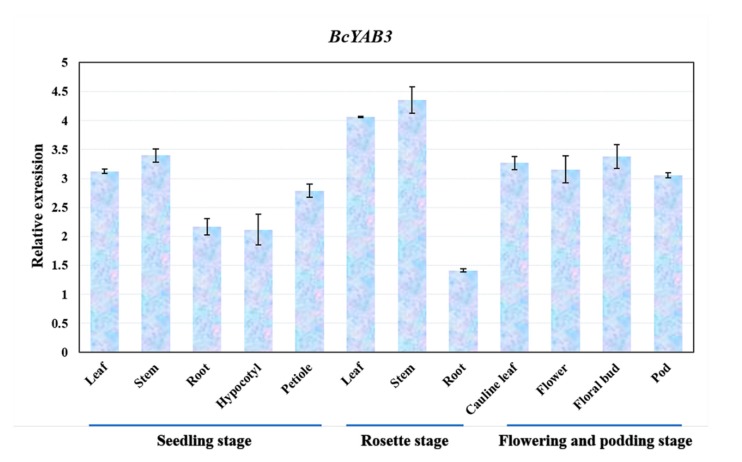
Expression patterns of *BcYAB3* in different tissues. The expression levels of *BcYAB3* with qRT-PCR amplification in leaf, stem, root, petiole, hypocotyl, flower bud, flower, and pod. The data represent the means of three replicates, and error bars represent the standard deviations of means.

To understand the expression patterns of *BcYAB3*, samples of different tissues in three developmental growth stages of Pak-choi were collected and identified using a qRT-PCR assay. The result showed that *BcYAB3* was expressed in diverse tissues and had a high expression in stems and leaves, followed by floral organs, but low expression in roots and the hypocotyl (Figure 3). This result is consistent with the function of YABBY genes in SAM and leaf development in previous studies.

### 3.3. BcYAB3 Shows Nuclear Localization

**Figure 4:**
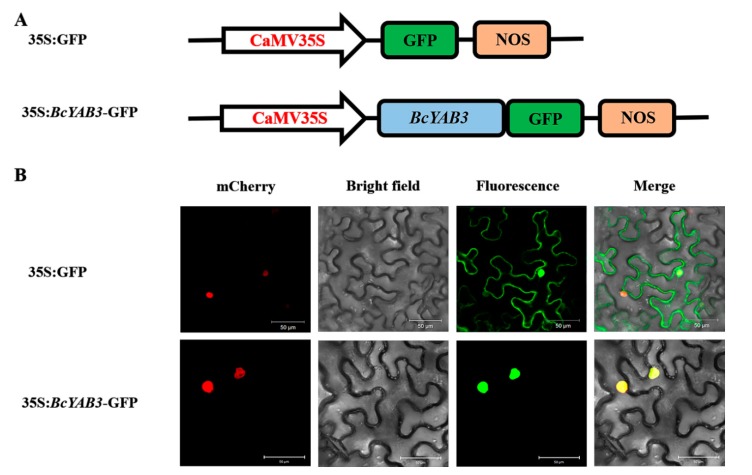
Subcellular localization of BcYAB3. (**A**) The construct of 35S:GFP and 35S:*BcYAB3*-GFP. GFP: green fluorescent protein; NOS: nopaline synthase gene. (**B**) Transient expression of 35S:*BcYAB3*-GFP fusion protein in tobacco. 35S:GFP was used as a control. Fluorescence images of mCherry (a nuclear marker), GFP, and merged were captured with confocal laser scanning microscopy and are displayed in red, green, and yellow, respectively. Scale bars = 50 um.

To verify whether the expression of *BcYAB3*, as a putative transcription factor, is localized in the cell nucleus, the 35S:*BcYAB3*-GFP recombinant construct was generated and transformed into tobacco epidermal cells through the *Agrobacterium* injection method. Under a laser scanning confocal microscope, the green fluorescence of the 35S:*BcYAB3*-GFP fusion protein was observed and localized specifically in the nuclei, whereas the protein of 35S:GFP was found to be expressed in both the nucleus and the cytoplasm (Figure 4). The results demonstrated that *BcYAB3* is a nuclear-localized protein.

### 3.4. Ectopic Expression of BcYAB3 Resulted in Pleiotropic Phenotypes in Arabidopsis

To investigate the function of *BcYAB3*, we firstly transformed *BcYAB3* into *Arabidopsis* for overexpression. In this study, a total of thirteen transgenic lines (named as OE1–13) were acquired after screening by PCR amplification and qRT-PCR assay (Figure S2). Among them, seven transgenic lines with relatively higher expression levels were selected for further analysis. The overexpression lines of *BcYAB3* could be classified into two types according to the phenotype observation. 

**Figure 5:**
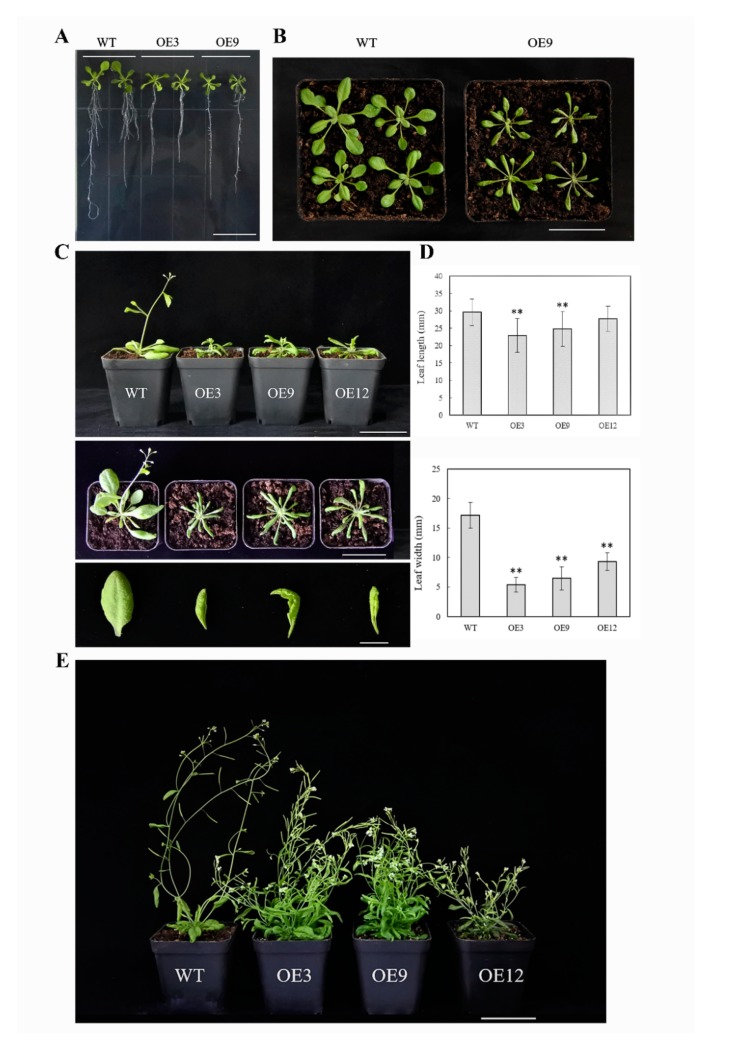
Type I phenotype of 35S:*BcYAB3*–GUS transgenic plants in *Arabidopsis thaliana.* (**A**) Fourteen days of *BcYAB3* transgenic plants grown on Murashige and Skoog (MS) medium with hygromycin. Scale bar = 2 cm. (**B**) The leaves of 22-day-old seedlings. Scale bar = 5 cm. (**C**) Phenotypes of the whole plant and leaves during bolting stage. Scale bars indicate 5 cm and 1 cm, respectively. (**D**) Leaf length and width statistics of the rosette leaves of 25-day-old plants. Error bars represent standard deviation of the mean number of 25 plants for each line. ** means significant differences compared to wild type (*P* < 0.01). (**E**) Phenotype of *BcYAB3* and wild type plants during flowering and fruiting stage. WT: wild-type *Arabidopsis thaliana*; OE3/9/12: transgenic lines of *BcYAB3*. Scale bar = 5 cm.

In type I, which had a normal growth and development, the transgenic plants (OE3, OE9, OE12) were relatively dwarf compared to the wild type (Figure 5). In the seedling stage, the root system with fewer lateral roots was weaker than the wild type (Figure 5A), the leaves were mildly curled, and then became long–narrow and significantly curled downwards with plant growth (Figure 5B,C). The bolting and flowering stages were delayed; the height of the bolt was shorter than the wild type, while the number of floral axes and leaves increased tremendously. There was no obvious main stem in transgenic plants, and the plants were capable of bearing fruit. 

**Figure 6:**
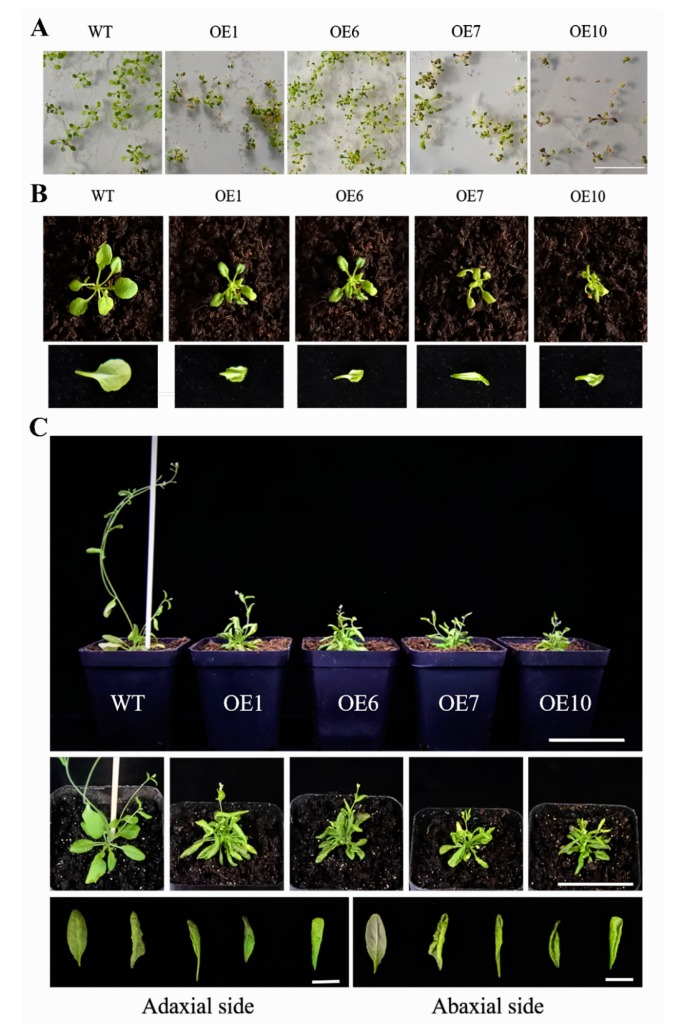
Type II phenotype of transgenic *Arabidopsis* with *BcYAB3* gene. (**A**) Phenotype of 10-old-day seedlings. Scale bar = 2 cm. (**B**) Phenotype of 18-old-day seedlings and characteristic of abaxial side of leaves in wild type and transgenic plants. Scale bar = 1 cm. (**C**) Phenotype of transgenic plants and wild type during flowering and fruiting stages. The phenotype was observed in a front view and top view, respectively. Scale bars = 5 cm. Comparison of the adaxial and abaxial sides of the leaves of wild type and transgenic plants. Scale bar = 1 cm. WT: wild-type *Arabidopsis thaliana*; OE1/6/7/10: transgenic lines of *BcYAB3*.

Type II, which had a severe phenotype, exhibited a diminutive and bushy plant phenotype (Figure 6). The leaf blades were clearly smaller than the wild type; the degree of downward curling of leaves gradually increased with the development of plants. The rosette leaves clustered together, and the flower stalks were shorter than the wild type at bolting and flowering stages; the height of transgenic plants was markedly decreased, and the ability to bear fruit was largely suppressed. In addition, a small number of purple seedlings were found in transgenic plants (Figure 6A, OE1/OE7/OE10), which may be caused by the accumulation of anthocyanins [14]. These results revealed a pivotal contribution of *BcYAB3* in leaf development; moreover, the overexpression of *BcYAB3* in *Arabidopsis* prolonged the vegetative period and delayed the flowering stage.

## 4. Discussion 

Several lines of evidence show that YABBY genes have a general role in the development of the meristem [28,41], leaves [30], floral organs [42], and fruits [43]. In *Arabidopsis*, the YABBY gene family plays a pivotal role in the development of abaxial cell fate in lateral organs. Numerous YABBY genes involved in diverse processes of growth and development were also identified and studied in other plant species [14,16,44,45]. Nevertheless, the functions of Pak-choi YABBY genes in plant development have not yet been revealed. *BcYAB3*, belonging to the *FIL*/*YAB3* subgroup, is homologous to YAB3 in *Arabidopsis*, which is related to leaf polarity, implying that *BcYAB3* possibly has a similar function in the regulation of leaf development. We profiled the transcript level of *BcYAB3* in various tissues at three developmental stages, including leaves, stems, roots, petioles, hypocotyls, floral organs, and pods. The qRT-PCR results indicated that *BcYAB3* was highly expressed in stems and leaves, especially in the rosette stage, followed by floral organs, suggesting that *BcYAB3* possibly participates in the regulation of leaf and floral development. 

Previous reports showed that *FIL*, *YAB2*, *YAB3*, and *YAB5* genes were associated with the development of vegetative tissues in *Arabidopsis*, while *INO* and *CRC* were involved in the ovules and carpels [10,19,46]. Moreover, the function of YABBY genes in tomato, *Antirrhinum*, and cabbage is similar to the *Arabidopsis* YABBY genes [15,37], indicating that YABBY genes have a conserved function in dicotyledonous plants. However, YABBY genes have different roles in monocotyledons, such as maize, rice and wheat [23,24,25,36,47], shown by the functional divergent phenomena YABBY genes produced in monocotyledons and dicotyledons. In this study, an unrooted phylogenetic tree was constructed and exhibited an evident separation between monocotyledons and dicotyledons, which was consistent with previous studies. Thus, the roles of Pak-choi YABBY genes can be speculated based on the results of previous research. 

To further investigate the function of *BcYAB3* gene, we firstly transformed the *BcYAB3* gene into *Arabidopsis*, and thirteen transgenic lines were obtained via screening and verification of PCR amplification and GUS staining. In comparison with wild-type plants, the leaves of transgenic plants with 35S:*BcYAB3*–GUS exhibited outward curled cotyledons, and then became long–narrow and dramatically curled towards the abaxial side with increases in leaf age. Measurement of leaf traits with 25-day-old seedlings showed that the width of transgenic *Arabidopsis* plants was significantly shorter than that of the wild type. This phenotype has a similarity with *Arabidopsis* overexpressed *AtYAB3* to some extent, which produced epinastic and narrow leaves. However, in most cases, the SAM of transgenic seedlings expressing ectopic *AtYAB3* was disrupted and only produced cotyledons [14]. In this study, the transgenic plants overexpressed *BcYAB3* produced cotyledons and leaves in both type I and type II, suggesting the functional difference of YAB3 gene existed in different species. Furthermore, the bolting and flowering stages were delayed in transgenic plants with 35S:*BcYAB3*–GUS, the inflorescence rachis increased, and leaves clustered together during the flowering stage. Conclusively, these data confirmed that *BcYAB3* plays a crucial role in specifying abaxial cell fate in lateral organs originated from apical and flower meristems.

## 5. Conclusions

In summary, this study provides first insights into the function of the Pak-choi *BcYAB3* gene and showed its implications in the development of leaves. This study paves the way for future research on YABBY genes to understand the regulatory mechanism of leaf polarity in *Brassica* species. Furthermore, the function of BcYAB3 in delaying the flowering stage will contribute to vegetable crops to prolong the harvest period for improving crop yield and achieving the objective of long-term market supply.

## Supplementary Materials

The following are available online at https://www.mdpi.com/2073-4425/11/4/370/s1, Table S1: Sequences of YABBY proteins used in this study. Table S2: Primers used in this study. Figure S1: The secondary structure of *BcYAB3*. Figure S2: The verification of T3 positive transgenic plants.
