# Supplementary material for: Ectopic Expression of a Pak-choi YABBY Gene, BcYAB3, Causes Leaf Curvature and Flowering Stage Delay in Arabidopsis thaliana
Source: Genes (Basel). 2020 Mar 29;11(4):370. doi: 10.3390/genes11040370 (PMC7230965; doi:10.3390/genes11040370)
Supplement: Supplementary file 1 [file genes-11-00370-s001.zip › Supplementary Figures' Lenges.docx]

Figure S1 The secondary structure of *BcYAB3*.

Figure S2 The verification of T3 positive transgenic plants.

**(A)** The construct of 35S:*BcYAB3*-GUS.

**(B)** The PCR amplification of *BcYAB3* using gene-specific primers. M: DL2000 DNA Marker; ‘ + and – ’ means positive plasmid of *BcYAB3* and ddH2O were used as PCR template, respectively. **(C)** qRT-PCR examination of BcYAB3 in wild type and transgenic plants.

Table S1 Protein sequences of YABBY genes used in this study.

Table S2 Primers used in this study.
