# Supplementary figures and images for: Ectopic Expression of a Pak-choi YABBY Gene, BcYAB3, Causes Leaf Curvature and Flowering Stage Delay in Arabidopsis thaliana
Source: Genes (Basel). 2020 Mar 29;11(4):370. doi: 10.3390/genes11040370 (PMC7230965; doi:10.3390/genes11040370)

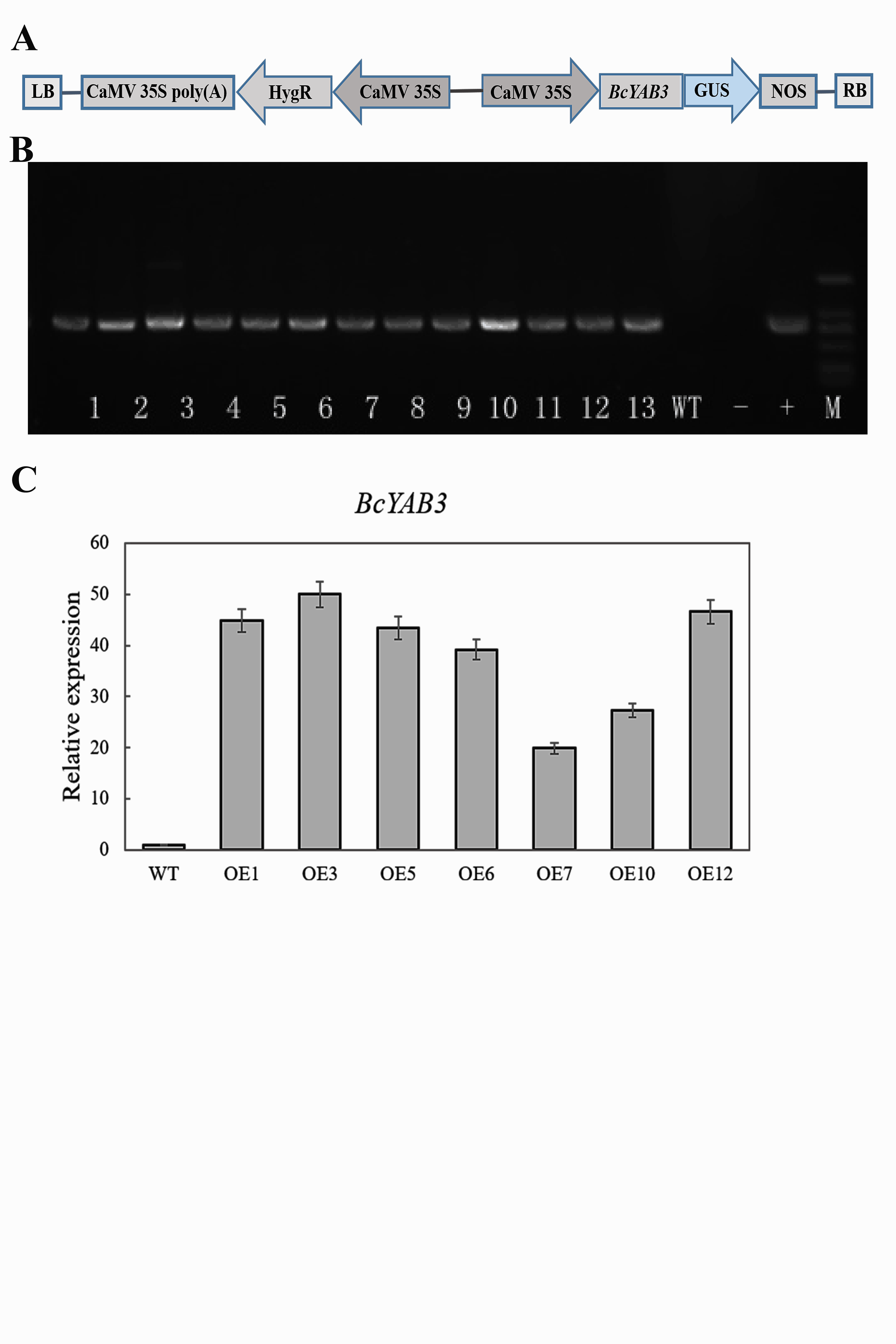

Supplement: Supplementary file 1 [file genes-11-00370-s001.zip › Figure S2.tif]
